# Physicians’ required competencies in AI-assisted clinical settings: a systematic review

**DOI:** 10.1093/bmb/ldae025

**Published:** 2025-01-16

**Authors:** Lotte Schuitmaker, Jojanneke Drogt, Manon Benders, Karin Jongsma

**Affiliations:** Department of Bioethics & Health Humanities, University Medical Center Utrecht, Utrecht University, Universiteitsweg 100, 3584 CG Utrecht, the Netherlands; Department of Bioethics & Health Humanities, University Medical Center Utrecht, Utrecht University, Universiteitsweg 100, 3584 CG Utrecht, the Netherlands; Department of Neonatology, University Medical Center Utrecht, Utrecht University, Heidelberglaan 100, 3584 CX Utrecht, the Netherlands; Department of Bioethics & Health Humanities, University Medical Center Utrecht, Utrecht University, Universiteitsweg 100, 3584 CG Utrecht, the Netherlands

**Keywords:** artificial intelligence, medical AI, AI ethics, digital health, AI competencies, systematic review

## Abstract

**Background:**

Utilizing Artificial Intelligence (AI) in clinical settings may offer significant benefits. A roadblock to the responsible implementation of medical AI is the remaining uncertainty regarding requirements for AI users at the bedside. An overview of the academic literature on human requirements for the adequate use of AI in clinical settings is therefore of significant value.

**Sources of data:**

A systematic review of the potential implications of medical AI for the required competencies of physicians as mentioned in the academic literature.

**Areas of agreement:**

Our findings emphasize the importance of physicians’ critical human skills, alongside the growing demand for technical and digital competencies.

**Areas of controversy:**

Concrete guidance on physicians' required competencies in AI-assisted clinical settings remains ambiguous and requires further clarification and specification. Dissensus remains over whether physicians are adequately equipped to use and monitor AI in clinical settings in terms of competencies, skills and expertise, issues of ownership regarding normative guidance, and training of physicians’ skills.

**Growing points:**

Our review offers a basis for subsequent further research and normative analysis on the responsible use of AI in clinical settings.

**Areas timely for developing research:**

Future research should clearly outline (i) how physicians must be(come) competent in working with AI in clinical settings, (ii) who or what should take ownership of embedding these competencies in a normative and regulatory framework, (iii) investigate conditions for achieving a reasonable amount of trust in AI, and (iv) assess the connection between trust and efficiency in patient care.

## Introduction

The use of Artificial Intelligence (AI) in clinical settings may offer great benefits [1–4]. It is argued that AI can reduce costs, increase efficiency, and improve clinical decision-making and patient outcomes [1,2]. Additionally, it is suggested that medical AI may equal or exceed the performance of medical specialists in certain medical tasks [1–4].

Yet, the integration of AI into clinical care remains challenging, due to barriers to the successful implementation of AI in clinical settings and routine patient care [5,6]. These barriers include unresolved questions about how AI may impact clinical decision-making, particularly regarding the physician’s role [2,4,7–11]. Increasing digitalization and AI usage raise the question which aspects of the medical profession are to be carried out by a physician, including what competencies will or will not remain important to the profession and what is needed to deliver good quality care [2,7]. Furthermore, questions remain about AI’s impact on the patient-physician relationship [2,11]. Some suggest that AI will take on a prominent role within this relationship, perhaps prominent enough to turn this dual relationship into a triad one: the patient, physician, and AI [11]. Relatedly, these potential developments raise concerns about the influence of AI on patient autonomy, the need for transparent reporting on the use of AI, and overreliance on AI [10,11].

To responsibly translate AI’s potential from bench to bedside, insights into the requirements for adequate functioning of medical AI are paramount. This is illustrated by the fact that, so far, the design and performance of the AI model are often key subjects of interest [12]. Nevertheless, for the responsible implementation of AI in clinical settings, it is equally important to gain insight into the requirements of human users [13]. After all, regardless of the quality of the AI application, without adequate skills and competencies of the physicians, clinical outcomes may not be improved, or outcomes may even deteriorate [14].

Currently, despite its importance for the successful implementation of AI in clinical settings, an overview of the human requirements required to adequately use AI at the bedside is missing. In this article, we, therefore, provide an overview of the academic literature with respect to the implications of medical AI for the required competencies of physicians. In this context, we specifically address considerations regarding physicians’ competencies in light of the patient-physician relationship in AI-assisted clinical settings. Additionally, we elaborate on normative aspects of AI in clinical settings affecting the competence-related dynamics at the bedside when AI is used in clinical settings.

## Method

To identify an overview of the required competencies of physicians when using medical AI in clinical settings, we have conducted a systematic review of the academic literature. Required competences are addressed with different epistemic terms, such as necessary knowledge, attitudes, and skills, we adopt a broad definition of *competency* in our review. We specifically adopt Morales-Sánchez and Cabello-Medina’s definition, namely ‘competencies are generally understood as a set of knowledge, attitudes, skills, and abilities that allow for individual excellence in the performance of a certain task or job’ [15]. Furthermore, competencies can be reflected by observable behavior, where virtues and responsibilities are often regarded as character traits [15]. The Preferred Reporting Items for Systematic Reviews and Meta-Analyses (PRISMA) guidelines for systematic reviews and the adapted PRISMA ethics guidelines were followed throughout the reviewing process [16].

### Search strategy

A literature search was conducted using PubMed, Embase, and Web of Science databases to find relevant articles on the required competencies, including skills, virtues, and responsibilities, of physicians using medical AI. This search strategy was discussed with an experienced librarian of the University Medical Center Utrecht, including the use of the appropriate databases, the design and structure of the search strings, and the relevance of the used terms and keywords. The initial search was performed on March 1st 2023 and a second search was conducted on September 17th, 2023 (see Supplemental File 1 for the search string).

### Article selection and inclusion criteria

Peer-reviewed academic articles that discussed the needed competencies, skills, virtues, or responsibilities for physicians using medical AI, were written in English or Dutch and published in 1979 or later, were eligible for inclusion. Articles that are not academic, not about medical AI, not about the physician’s roles and skills, or not about the needed skills, virtues, competencies, or responsibilities of physicians were excluded.

Articles were selected for eligibility based on the screening of titles, abstracts, and keywords. Two researchers independently screened the titles and abstracts (LS and KJ) and, if applicable, the full texts of the articles (LS, JD, KJ). In case of disagreement about inclusion or exclusion, differences were discussed until a consensus was reached. The reference lists of included articles were subsequently screened for additional relevant articles.

### Data extraction and analysis

The selected articles were analyzed using a data extraction form in which keywords, key messages, and overarching themes of the text were extracted in a systematic way. As such, excerpts about the needed skills of physicians for using AI, visions about the responsibility of physicians using AI, and normative aspects of AI in care were extracted. These excerpts were subsequently compared and categorized into three overarching themes. Within these overarching themes, the information was bundled into more acuminated subthemes.

## Results

**Figure 1:**
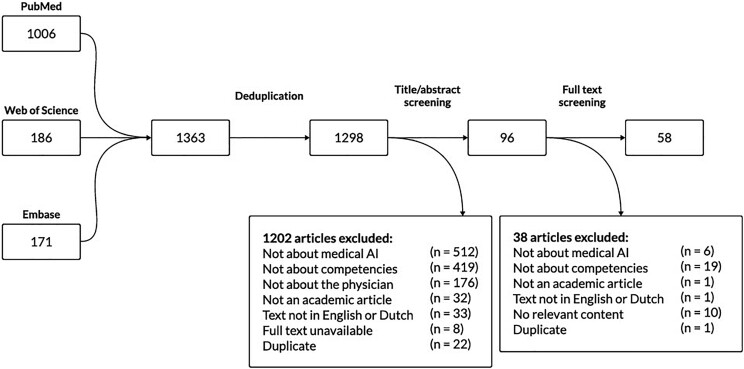
Flowchart of article selection, screening, and inclusion.

A total of 1298 articles were retrieved from the databases and included for title and abstract screening. After title and abstract screening and full-text screening, 58 articles were included for data extraction and analysis (Fig. 1).

The results are categorized into three overarching themes. The first theme contains all considerations regarding the competencies physicians need to work (more) adequately with AI in clinical settings. The second theme provides a comprehensive overview of the literature regarding how physician competencies may be affected by the (changing) roles of physicians in the patient-physician relationship amidst the rise of medical AI. The third theme focuses on the state of the literature regarding key normative and regulatory aspects of working with AI in clinical settings that impact the ability of physicians to become competent AI users. These aspects include an overview of what is argued about responsibility, regulations—or the absence thereof—and trust. In the following sections, these themes are discussed in more detail.

### Theme 1: physicians’ required competencies when using medical AI

#### The need to develop digital competencies

A frequently mentioned requirement was training physicians to improve their digital and technical skills [17–24]. First, physicians need to have a basic understanding of how data is aggregated, analyzed, and used in AI-mediated healthcare delivery [22,25–32]. The required competencies include big data use, statistics, and information management. Some specifically argued that, besides these skills, increased knowledge about the technology underlying AI and competence in using AI systems are needed [17–24], including the ability to evaluate its output, assess its significance, and detect errors of AI-assisted medicine.

Second, medical education should be tailored to these digital demands, meaning that students should be trained specifically on the fundamentals and technology underlying AI, core computer science, and technological adaptation [22,26,28,33]. Additionally, physicians and medical students should be trained to detect errors in AI-assisted medicine [17,18]. Nevertheless, the extent to which medical practitioners need to grasp AI’s inner workings varied, which was illustrated by studies mentioning that it may not be strictly necessary to fully understand every aspect of the black box underlying AI in decision-making, as long as the outcomes of such decision-making processes can be validated and, therefore, relied upon [34–36]. On a similar note, it was mentioned that several technical and procedural skills used by physicians in diagnosis and surgery can become less important and may be carried out by AI-driven tools [37]. However, some have warned that physicians should not merely become technicians who blindly follow AI’s orders [35,38,39].

Third, physicians are expected to have the competencies to interact more with technical fields and experts outside the medical domain, such as AI specialists and data scientists [21,25,32,38]. Some took a stronger stance, suggesting that physicians should collaborate with non-medical digital specialists and give them an official role in their medical teams [27,40].

Fourth, it was suggested that new areas of medical expertise may emerge, creating new jobs and areas of expertise or subspecialties in medicine that require additional (digital) competencies [40,41]. Examples of such subspecialties may be medical data scientists or medical AI specialists.

#### Fostering intrinsic human skills

Many authors underlined the relevance of non-technical skills in the age of digital health technologies and AI [18,19,24–26,30,37,42–46]. Repetitive and administrative tasks could potentially be delegated to AI appliances [21,24,25,27,33,34,41,44–54]. As medicine is believed to become increasingly ‘high tech', human or ‘soft’ skills should play a more prominent role in physicians’ work [25,37,45,47,49,55,56].

Moreover, it was argued that AI will never be able to fully master, and therefore replace, the so-called ‘critical human skills’ that are needed to adequately perform the complex process of providing effective care and clinical judgement [25]. For example, giving adequate care requires subtle emotional intelligence, the ability to communicate with the patient, empathic listening, situational judgement, interpreting vague signs and tone of voice, making eye contact, and signaling vague discomfort [18,19,24–26,42–44]. In addition, it was argued that clinical judgement is a process that requires multiple intrinsically human elements, including empathy, motivations, desires, wisdom, and character [19,45]. Some have argued that critical human skills should have priority in medical curricula and medical training in general [19,22,25,26,28,33].

Additionally, it was argued that practical human skills, such as performing surgery, are central to the medical profession [55]. These can arguably never solely be carried out by AI, and such skills will thus remain important for physicians.

#### Training physicians in AI ethics

Aside from the importance of training physicians in the use of technology in practice and in critical human skills, they must also become conscious users of AI in medical practice [19,23,25,26,37,57]. Therefore, it was argued that increased emphasis on critical thinking and ethical reflection regarding AI—and digital appliances in general—in medical practice is needed.

First, some authors stressed the need to inform physicians about ethical challenges that arise alongside the introduction of AI in the medical context [22,23,40,41,58]. Critical, problem-oriented thinking and the ability to combine information from multiple sources will gain importance, while the emphasis on factual knowledge will move more to the background. Without further clarification or (practical) examples, it was thus argued that skills and knowledge of ethical problems and complex issues regarding AI are essential [21,32,59]. Furthermore, some specifically voiced a sense of urgency to educate (future) physicians on the limitations and pitfalls of such systems, in addition to the strengths and possibilities [29,52].

Second, it was argued that physicians must be trained to understand the values built into AI-driven technology that may result in bias and racial discrimination patterns [22,23,41,60]. Ultimately, such technological and ethical literacy was said to be important for applying AI responsibly in medical settings and thus preventing harm [23,27,29,40,52,58].

#### Fear of skill erosion

aside from pressing concerns with regard to the required competencies for physicians who work with AI at the bedside; some authors also have expressed the concern that introducing AI in clinical practice may lead to an erosion of physician’s skills, i.e. deskilling, and a devaluation of the profession when skills are no longer regularly used [20,27,44,48,53,54,59,61]. This includes a loss of intellectual capacity and practical expertise [43]. Concerns of overreliance on AI, dehumanization, and loss of practical skills were also mentioned [20,32,44,45]. Some argued that this may result in eroded patient-physician trust [59], decreased diagnostic accuracy [48], or disruption of performance when technology breaks down [20]. Physicians may, for instance, lose skills due to automation and be unprepared in unanticipated crisis-like situations, similar to how pilots are too dependent on the autopilot function of an airplane [53]. Also, the fear of ‘alert fatigue’ was described, including situations where physicians become numb to the notifications of digital appliances, resulting in frustration [55]. Moreover, dependence on AI and laziness were mentioned [55], including its importance for physicians to remain aware of the risks to patient safety that such skill erosion may bring and the need to develop effective measures to mitigate this risk [53]. Also, it was argued specifically that physicians should maintain, at the very least, the same level of practical skills, to mitigate possible damages caused by AI [18].

#### Human irreplaceability: why physicians’ competencies matter

With the introduction of AI in clinical settings, there is speculation about the physician’s role and whether their competencies, or even they themselves as physicians, are still needed. Yet, the irreplaceability of the human physician was strongly emphasized [3,19,21,25,26,29,32,35,43,45–47,55,56,62,63], as physicians and AI combined can arguably augment clinical decision-making altogether and, as such, enhance the quality of care [29,32,49,55,61,62].

Insofar as authors elaborated why human-AI combinations are desirable, it was firstly argued that the physician’s relief in workload due to AI is one of the main reasons for the increased emphasis on the ‘human’ aspect of physicians [45,55,56]. Furthermore, it was argued that we should not seek the potential of medical AI in the simulation of human capabilities but rather in the progression of how these human capabilities can be realized [29]. Also, it was argued that while the use of AI can undoubtedly benefit the quality of care, it can never replace critical components of the patient-physician relationship, such as trust, loyalty, and empathy [45,55,56]. Only human physicians can practice ‘the art of medicine’ [18]; and only human physicians can genuinely care for the patient [3].

Second, it was argued that the capabilities of AI applications in health care stretch only so far as working at a specific task level because the complete job of a physician requires a more sophisticated set of skills [3,47]. Furthermore, successful medical AI relies on following repetitive patterns and some form of methodological replicability, but human patients can rarely be understood in such methodological and replicable ways [3,18,32,55]. Therefore, clinical reasoning cannot be completely deduced to repetitive patterns or merely following a set of rules; every consultation is unique, and it was argued to be impossible for an AI to do this adequately [3,18,46,55]. This distinctive limitation of AI, when implemented at the bedside, leads to the emphasis on the importance of a skilled human physician at the bedside to conduct proper clinical reasoning [3,18,55]. The ability to conduct clinical reasoning requires experience, sensitivity to context, and intuition [3,18,46,55].

Third, it was argued that good quality care relies on interpersonal relationships and human warmth specifically [45]. Furthermore, as there is no such thing as artificial empathy, empathy was considered to be important for good quality caregiving [3,29]. In the same line, it was proposed that people want to be ‘cared for’ by humans, as actions can be stronger than words: a facial expression of sorrow or a hand on the shoulder of the patient can be meaningful elements of the patient-physician relationship [3,18]. Furthermore, it was argued that understanding the patient’s needs can only be done by humans [46].

Last, the ability to explain one’s reasoning is essential for a physician and something that AI can never do in the same way [55]. Likewise, it was argued that AI systems require specific input from humans, and their output needs evaluation from humans specifically [21].

### Theme 2: the impact of shifts in the patient-physician relationship on physicians’ competencies

#### The gift of time: gaining the time to care

The extent to which physicians must apply particular competencies and take on their role within the patient-physician relationship may change when AI becomes more prominent in clinical settings. First, this may be due to a shift in the type of tasks of the future physician. As mentioned above, it was argued by many authors that AI in clinical settings can be used to take over low-cognitive repetitive administrative tasks that were traditionally part of the physician’s profession [25,37,45,47,49,55,56]. This will likely improve the efficacy of clinical practice without compromising patient safety and may, as a result, bring physicians ‘the gift of time’ in the context of the patient-physician relationship, meaning that the physician can spend more time with the patient [21,24,25,27,34,45,46,48–50,53,56,64]. In turn, it was argued that time saved in certain aspects of clinical practice may increase the value of the physician and place the focus more prominently on other areas of caregiving [27,48,49,56]. These areas include tasks that depend on competencies and skills that have traditionally been central to the medical profession but could not always be carried out due to time constraints, such as interaction and communication with the patient [27,48,49,56]. It was argued that this can subsequently result in better quality care, for instance, by contributing to a patient-centered medical paradigm, where the administrative burden of physicians will make room for more interaction with the patient [27,49].

#### Shared decision-making in AI-assisted clinical settings

Since a typical day in the life of a physician may change when AI is implemented in clinical settings, it can be expected that the patient-physician relationship will change too, also in the context of shared decision-making [17,19,22,45,52,64,65]. As such, the role of the physician and their subsequent required competencies may undergo changes. First, shared decision-making dictates that physicians inform patients with the best available evidence and provide each patient with adequate support to consider their options and arrive at an informed decision [47]. It was argued that AI-mediated appliances in the physician’s clinical toolbox add to this evidence base [19,22,47,64,65]. Consequently, it will be the task of the physician to explain, interpret, or translate the functioning of the AI model when it is important for the patient to make an informed decision if need be. This can, for instance, be compared to how physicians ought to inform the patient about possible side effects of treatments: they have to be able to explain to patients when predictions have been made based on AI and what that means in their specific context [45]. Moreover, patients may expect a physician to be able to evaluate the significance of the output of AI, compare it with their own professional judgement, and relate this to the patient’s preferences, expectations, or concerns [28,52,64]. It was argued specifically that the function of the physician in such settings is to ensure informed consent, autonomy, and protection from harm [17,52].

Second, it was argued that physicians should take an active role in ‘translating’ the abundance of information, for instance, the output of AI appliances in clinical settings, in an understandable manner to the patient [17,22,42]. To this end, they should be trained in effective and empathic communication with patients [21,29,41]. Physicians will be expected to remain, or increasingly be, the patient-facing actor when the role of AI becomes more prominent in the decision-making process. Such a patient-facing actor was considered important because that the AI-driven automation of some processes in clinical settings may be at the expense of the time patients and physicians spend together [26].

Third, it can be expected that there will be a shift in terms of access to information about health and illness—whether it be general information or specific information on people’s own health status. Information that was once exclusive to physicians is now much more accessible to the public [37]. Patients can, for instance, more easily self-diagnose or monitor their own well-being [37,41]. Without further clarification, some argued that physicians should be trained to handle this information-shift within the patient-physician relationship [66].

Fourth, it was argued that the black-box nature of certain AI appliances may hamper the process of shared decision-making because of the opacity underlying AI-generated clinical recommendations and, therefore, can never articulate this adequately to the patient [40]. Some have also expressed the fear that AI will diminish the role of the physician in the process of shared decision-making [49], since they expect patients to rely on nearly autonomous decision-making by AI without a human in the loop. This can arguably be a challenge for responsibility and accountability. Moreover, paternalism may be reintroduced when patients blindly follow suggestions made by either an AI system or a physician who follows suggestions driven by AI [39].

#### Preserving epistemic authority

It was argued that the deployment of medical AI may challenge the epistemic authority of physicians within the patient–physician relationship, especially in cases when the physician and the algorithm ‘disagree’ on the best course of action [48,60]. Traditionally, when two equally experienced physicians disagree with each other, they are trained to explain their reasoning to one another and possibly detect each other’s errors and blind spots. Conversely, when the physician and algorithm disagree, it is unclear how much weight the suggestion of the algorithm carries. Also, an algorithm can never adequately justify its line of reasoning to physicians and other relevant addressees, such as the patient or authorities [31,55,60]. Furthermore, it was argued that algorithms will never have the epistemic authority to be an equal ‘sparring partner’ for a physician, which renders the physician ultimately responsible for clinical decision-making [60]. Also, it was argued that the opaque and black-box nature of AI could make it unclear to whom responsibility should be delegated [48]. Moreover, it was suggested that physicians should thus be trained in managing such human-AI disagreements [48].

Despite these challenges, the majority of the authors argued that physicians will maintain their epistemic authority and remain responsible for the consequences of their actions, even though some—or many—of the clinical tasks are delegated to AI [21,22,31,33,34,40,45,46,55,60,61,64,66,67]. Some presented this argument by comparing medical AI usage with self-driving cars, arguing that physicians will remain responsible for using AI in the same way that drivers remain responsible for using self-driving cars [33].

The explanations as to why physicians should remain responsible, insofar as provided, varied. It was, for instance, argued because of ‘psychological reasons’, meaning that physicians simply want to stay in control for no particular and objective reason [55]. Another reason had to do with the professional autonomy of physicians; without being the ones making the final decisions, professional autonomy would be ignored, and that would be undesirable in itself [55]. Moreover, a lack of responsibility would make the profession less ‘enjoyable’ [55].

### Theme 3: key normative and regulatory factors underlying physicians’ competence in AI-assisted clinical settings

#### The need for a proper regulatory framework for working with AI

In addition to functioning as prerequisites for physicians to utilize and develop required competencies, normative and regulatory factors may also determine whether physicians can use AI adequately in clinical settings. These include a proper regulatory framework that addresses accountability, responsibility, and trust [26,38,40,41,49,50,52,54,63,64,67–70]. To this end, it was argued that the role and responsibility of physicians should be made clear in legislation and (soft) law. Although nearly all the authors refrained from elaborating more in-depth on the content of such regulations, some did specifically underline the pressing existence of a knowledge gap in relation to responsibility in clinical decision-making mediated by AI [64,69].

First, such regulations should, at the very least, clarify the risks of AI usage in medical settings and ensure legal security for all actors involved to safeguard trust among medical professionals [64]. The opaque nature of (some of the) AI appliances in medical settings makes it, however, impossible to fully assess risks beforehand or prove wrongful action afterward, which hampers such legal security [64]. Others suggested that, for orientation on an adequate and ethical and legal framework for the usage of AI in medical settings, the four principles of medical ethics by Beauchamp and Childress [64,71], the principles of ‘good medical or scientific practice’, [40] or the ‘traditional’ principles of good care [52] can be used as a blueprint. Also, it was argued that the legal framework of AI in clinical practice should incentivize physicians to use AI at the bedside, but in a way that overreliance on AI is prevented as much as possible [18]. Their suggestion was to make the standard of care so that AI can be used but always in addition to a review by a (human) physician for quality control. Humans should be responsible for identifying misbehavior, mitigating bias, preventing harm, and providing accountability [18,58].

Second, it was argued that, to create a proper regulatory framework, the key ethical and legal considerations of AI in clinical settings should focus more strongly on the potential risks and harms and less on the potential benefits of AI [40,58]. In line with this, it was argued that awareness of AI’s potential to disrupt our current understanding of professional responsibility and accountability is important while making rules and regulations in addition to its appropriate use [40].

Third, it was argued that the concept of responsibility considering medical AI should not be reduced to ‘respect for the rules’ once they are installed. Instead, professional training in the ethical use of medical AI needs to be at the forefront, in addition to clear guidelines [69].

Fourth, it was argued that guidance must be provided about what can and cannot be trusted with regard to AI in clinical settings [40]. Yet, while such a need for normative guidance was echoed frequently in the academic debate on the required competencies for physicians using AI at the bedside [26,38,40,41,49,50,52,54,63,64,67–70], concrete recommendations for such guidance were not made clear.

#### Becoming responsible for AI

Some have promoted the need for a bottom-up approach to responsible and equitable deployment of AI in clinical settings, where physicians themselves take active steps and feel responsible themselves [21,22,31]. Directives and regulations may hamper the intrinsic feeling of physicians to feel responsible for deployment in medical care [31]. Responsibility to ‘do good’ was thus placed on the physicians themselves [21,22]. Others argued in favor of a top-down approach, where medical professional organizations and policymakers should be the driving force of the debate on responsible AI implementation in clinical settings [41,52]. Both sides agreed that an active approach is needed; a laissez-faire approach without oversight or defined goals was considered unacceptable [22,52].

Of the authors arguing that physicians were the actors to carry responsibility, some stressed specifically that such responsibility can never adequately be assigned without adequate training in the proper use of AI [18,31,34,66] and empathic listening and communicating skills [44]. In contrast, some argued that responsibility does not necessarily rest on the shoulders of the physician [38]. They argued that responsibility depends on the level of control one has over the consequences, and that liability should be appointed depending on the level of control one has over AI in the process of clinical decision-making. This means that, in their view, liability can be appointed to the physician, but also to the AI developer, other healthcare providers, or the healthcare system [38]. Others argued that it is impossible for a physician to be held (legally) responsible for adverse outcomes in the clinical setting if AI is involved in the process of clinical decision-making [72]. The opaque nature of AI and the lack of control exercised by physicians over the output of AI systems would hamper this legal responsibility. These authors acknowledged that AI can only add value if it allows physicians to accept AI’s advice blindly, yet this will never be possible for the abovementioned reasons. To solve this dilemma, they stressed the importance of actively debating what level of epistemic uncertainty is acceptable under which circumstances [72]. In line with this, it was also argued that physicians should learn to better deal with uncertainty in light of AI in clinical settings [21].

#### Dealing with (dis)trust in AI

Given that it can be difficult to define or redefine trust in medical AI, the focus of some scholars has shifted to mitigating distrust in AI [36,69]. In that sense, it was argued that instead of longing for a solid definition of trust in AI, a more suitable concept would be to work towards physicians having ‘confidence’ in AI [69]. This means that a belief in the necessity that what has happened once will happen again (confidence) is more appropriate than to believe that something or someone can act in one’s interest (trust) [69].

Another notion of dealing with trust and distrust focused on the notion of uncertainty [36]. Here, it was argued that the concept of explainability, or the lack thereof in opaque AI systems, may carry too much weight in the debate on the implementation of AI in medical settings. It was argued that uncertainty has always been central to the profession. Everyday interventions, such as aspirin as a pain killer, were applied successfully regularly whilst their mechanisms were not understood. Therefore, the opaque nature and lack of causal insight of AI are not radically different from common aspects of medical decision-making; uncertainty is the rule rather than the exception. The need for physicians to be able to fully understand and explain the inner workings of AI appliances was therefore questioned [36].

Nevertheless, others denied the notion of trust altogether by arguing that trust in AI is, at this point, premature and that ‘tacit’ trust in medical AI may even be dangerous [7]. Tacit trust in AI can be understood in the same way as the tacit trust humans place in driving cars. Essentially, cars are computers on wheels with complex inner workings of such a level of complexity that transcends most of our intellectual capacity, yet we do drive them, assuming uncritically that the technology will work. Such an uncritical adoption of trust in AI in a medical setting would not only be premature but also dangerous [7].

Last, it was argued that a danger of physicians’ use of AI may be that it hinders the patients’ trust in them. Patients may view the physician not as a competent AI user, but merely as an intermediary between them and the digital system and lacking medical significance [44]. The more the physician relies on AI systems in the decision-making process, the greater the limits may be as to what the physician is able to explain about their decision. This affects patients’ trust in physicians [48]. Regulations and guidelines are needed specifically to ensure patient trust in physicians using AI [40].

## Discussion

This review presents an overview of the state of the literature with regard to the human requirements for competently utilizing medical AI in clinical settings. As such, we provide a comprehensive overview of the insights of the academic field regarding the physician’s required competencies, changes in the physician’s role in the patient-physician relationship, and normative and regulatory aspects of working with AI in clinical settings. It is, however, noteworthy that the descriptions of required competencies and prerequisites for competent usage were often minimal and ambiguous, with few suggestions for concrete guidance on how the competencies should be fostered. By providing a descriptive overview of the relevant aspects as mentioned in the literature, we thus offer a basis for subsequent further research and normative analysis.

Our review highlights the need to explore the appropriate balance between physicians developing digital competencies and nurturing essential non-technical human skills. Throughout this review, the contrast between the increasing need for technical and digital competencies, on one hand, and the emphasis on the non-technical side of medicine and human or soft skills, on the other hand, in AI-assisted clinical decision-making is noteworthy. It was mentioned frequently that physicians may need to upgrade their clinical toolbox to adequately navigate the increasingly digital medical landscape [17,18,21,22,26,28,29,33,39,41,42,45,47,60,66]. It was also argued repeatedly that, with medicine becoming more ‘high-tech’ itself, critical human skills and soft skills of physicians should have moved further towards the forefront in clinical decision making [18,19,24–26,30,37,42–47,49,55,56]. However, it remains still uncertain and ambiguous when and to what extent those competencies were applicable, as this can vary with medical contexts and specialization.

Furthermore, our review shows that it is essential to determine who or what should steer physicians’ training in required competencies, as well as assess the feasibility for physicians to allocate the time needed to acquire and demonstrate these competencies. Currently, there is no consensus on these topics, which we will also discuss in the following sections.

### How should physicians become responsible users of AI?

While there seems to be some consensus on the need for changes in the physician’s toolbox, or a change in the emphasis on their current competencies, such consensus is lacking about the question of who or what should trailblaze the pathway for these changes to be made. In other words, there remains ambiguity about who carries the responsibility to adequately equip physicians to navigate through the changing medical landscape. A logical place would be to alter the medical curricula in such a way that the physicians of the future are trained sufficiently. To this end, some suggestions on the contents of medical curricula were made, mainly with recommendations to supplement the curricula with more training on the fundamentals of AI and technology more broadly [22,26,28,33]. Yet, few concrete recommendations can be identified. Additionally, the issue of how to support practicing physicians persists.

A similar ambiguity remains about who should be at the forefront of the debate on the blueprint of a regulatory framework. What stands out is the fact that many acknowledge the importance of a normative and regulatory framework for AI in clinical settings, and that the lack thereof may hinder physicians from adequately implementing AI at the bedside [26,38,40,41,49,50,52,54,63,64,67–70]. Yet, there hardly seems to be consensus on who should take ownership of solving this predicament and what the way forward should be. Suggestions were made, and both the bottom-up and the top-down approaches were opted as promising [21,22,31,41,52]. Others prompted that ‘traditional’ principles of medical ethics can be used as inspiration for a proper framework of AI in clinical practice [40,52,64,71]. Yet, while suggestions were provided, few directions for practical guidance and adequate implementation of AI in clinical settings were given. This finding endorses findings of other ethicists in the field, who stress that inter-stakeholder cooperation at a policy level is needed to fit multiple AI-ethics agendas together, and to aim for procedural consensus on both ethical principles as the implementation of these principles in clinical settings [73].

### Allocating time to develop and demonstrate required competencies

One aspect that is not discussed thoroughly in the literature is that physicians need sufficient time to acquire and demonstrate required, especially novel, competencies. Interestingly, many seemed to assume that AI use will create more time for physicians, which they can dedicate to demonstrating non-technical qualities such as talking to the patient [21,24,25,27,34,37,45–47,49,50,53,55,56,64]. Nevertheless, it remains unclear how the efficiency gained by using an AI tool at the bedside will result in actual added (‘gifted’) time for the physician.

The ability of AI tools to increase efficiency and create more time for physicians seems to depend on underlying assumptions that were neither made explicit nor currently realistic. For instance, an underlying assumption appears to be that AI can perform specific bedside tasks without requiring doctors to spend extra effort verifying the tool’s results. Yet, for this to succeed, an AI tool must not only be proven accurate and effective, but it must also be reasonably trusted by the physician and other relevant stakeholders [74].

In other words, in order to increase efficiency, it is essential that physicians put a reasonable amount of trust in the AI tool and let it operate independently without having to (unnecessarily) ‘double-check’ the output generated by it. However, the notion of reasonable trust in AI is multifaceted and complex, especially when AI tools are opaque [10]. Furthermore, considering AI’s anticipated supportive role in clinical practice, the assumption of achieving such substantial efficiency improvements through AI appears unlikely at this time [75,76].

Given these barriers, it is even imaginable that, instead of giving the physician extra time, implementing AI in clinical settings may result in *more* work for physicians, for instance, by having to validate AI’s output or by AI being an extra step in the medical decision-making process that needs to be overseen by a physician [74]. In this case, the ‘gift of time’ as promised by the introduction of medical AI in clinical settings may ultimately proof to be the medical equivalent of a Trojan Horse, bringing unforeseen challenges alongside its apparent benefits. In future research, it would be valuable to focus on what conditions should be met to achieve reasonable trust in AI, especially given AI’s promise to increase efficiency and the importance of physicians developing certain competencies to adequately work with AI.

### Strengths and limitations

This systematic review provides a comprehensive overview of the academic literature about human requirements for medical AI. The articles presented were included after a thorough screening of the academic literature on the topic, based on a search strategy that was guided by experienced librarians. This has resulted in a review with useful insights for those working in the context of clinical settings. Nonetheless, the review has several limitations.

First, this review included data on circumstances in clinical settings that were deemed important. While the inclusion occurred systematically and with clear in- and exclusion criteria, this type of research is typically sensitive to reporting bias. Different groups of researchers may report different things as important and thus include or exclude different things. Second, while we were assisted by an experienced librarian to construct a comprehensive search query and strategy, it is possible that there are articles published that contain valuable information on the research topic, yet were not yielded by our systematic search query. For instance, because they described the topics of our interest in different wordings than our search string covered. Finally, we note that it was beyond the scope of this paper to assess the scientific validity of the aspects discussed in the included articles.

## Conclusion

This systematic review shows that there is consensus on the fact that the use of medical AI in clinical settings will influence how physicians, now and in the future, will carry out their profession and that they may need a wide range competencies to adequately use AI in clinical settings. At the same time, we found that descriptions of required competencies and prerequisites for competent usage were minimal and ambiguous, and few recommendations for concrete guidance were made. By offering a comprehensive overview of the state of the literature, this review gives direction for future areas of inquiry that address ongoing unclarity regarding physicians’ competencies and thereby contributes to fostering the implementation of AI at the bedside. With regard to physicians' competencies, the patient-physician relationship, and normative aspects of decision-making in clinical settings, this review highlights several essential directions for future research. Our review, for instance, highlights the need to explore the appropriate balance between physicians developing digital competencies and nurturing essential non-technical human skills. Additional attention is necessary for determining who or what should steer physicians’ training in required competencies. Also, it is important to assess the feasibility of physicians allocating the time needed to acquire and demonstrate required competencies and whether expected benefits like the ‘gift of time’ can actually be realized by introducing medical AI.

## Supplementary Material

Supplemental_file_2_Search_String_ldae025

## Data Availability

Articles retrieved from various databases were used to generate the results as laid out in the search string and flowchart for inclusion, selection, and screening. The search string and flowchart can be found in the supplemental files.

## Bibliographical details

The authors all work at the University Medical Center Utrecht and specialize in the ethics of new and emerging (bio)medical technologies, digital health, and medical AI [1]; and neonatology [2].
