# Supplementary material for: Physicians’ required competencies in AI-assisted clinical settings: a systematic review
Source: Br Med Bull. 2025 Jan 16;153(1):ldae025. doi: 10.1093/bmb/ldae025 (PMC11738171; doi:10.1093/bmb/ldae025)
Supplement: Supplemental_file_2_Search_String_ldae025 [file supplemental_file_2_search_string_ldae025.docx]

**PubMed**

(("Artificial Intelligence"[Mesh] OR "Artificial intelligence"[Title/Abstract] OR "Black box"[Title/Abstract] OR "machine learning"[Title/Abstract] OR "deep learning"[Title/Abstract] OR "self-learning"[Title/Abstract] OR "computerized clinical decision support system*"[Title/Abstract])

AND

("Medicine"[Mesh] OR "Medic*"[Title/Abstract] OR "Health*"[Title/Abstract] OR "healthcare"[Title/Abstract]))

AND

("Clinical Competence"[Mesh] OR "Clinical Competenc*" [TIAB] OR "virtues"[Mesh] OR "virtue*"[Title/abstract] OR "virtuous"[Title/abstract] OR "social responsibility"[Mesh] OR "responsibilit*"[Title/Abstract])

**Embase**

('artificial intelligence'/exp OR ‘Artificial intelligence’:ti,ab,kw OR ‘Black box’:ti,ab,kw OR ‘machine learning’:ti,ab,kw OR ‘deep learning’:ti,ab,kw OR ‘self-learning’:ti,ab,kw OR ‘computerized clinical decision support system*’:ti,ab,kw)

AND

(‘Medicine’/exp OR ‘medic*’:ti,ab,kw OR ‘health*’:ti,ab,kw OR ‘healthcare’:ti,ab,kw)

AND

('clinical competence'/exp OR 'clinical competenc*’:ti,ab,kw OR ‘virtue ethics’/exp OR ‘virtue*’:ti,ab,kw OR ‘virtuous’:ti,ab,kw OR ‘social responsibility’/exp OR ‘responsibilit*’:ti,ab,kw)

**Web of Science**

(TS=("artificial intelligence" OR "black box" OR "machine learning" OR "deep learning" OR "self-learning" OR "computerized clinical decision support system*"))

AND

(TS=(“medic*” OR “health*” OR “healthcare*”))

**AND**

**(TS=(“clinical competenc*” OR “virtue*” OR “virtuous” OR “responsibilit*”))**
